# Surgical treatment outcome of children with neural-tube defect: A prospective cohort study in a high volume center in Addis Ababa, Ethiopia

**DOI:** 10.1016/j.bas.2023.101787

**Published:** 2023-07-26

**Authors:** Abenezer Tirsit, Yemisirach Bizuneh, Bethelehem Yesehak, Mahlet Yigaramu, Asrat Demetse, Filmon Mengesha, Samuel Masresha, Eyob Zenebe, Samuel Getahun, Tsegazeab Laeke, Bente E. Moen, Morten Lund-Johansen, Rupavathana Mahesparan

**Affiliations:** a Division of Neurosurgery, College of Health Science, Addis Ababa University, Ethiopia; b Department of Clinical Medicine, University of Bergen, Norway; c Department of Gynecology and Obstetrics, College of Health Science, Addis Ababa University, Ethiopia; d Department of Pediatrics and Child Health, College of Health Science, Addis Ababa University, Ethiopia; e Department of Psychiatry, College of Health Science, Addis Ababa University, Ethiopia; f Departments of Global Public Health and Primary Care, University of Bergen, Norway; g Department of Neurosurgery, Haukeland University Hospital, University of Bergen, Norway

**Keywords:** Neural tube defect, Hydrocephalus, Surgical treatment, Outcome

## Abstract

**Introduction:**

Prevalence of neural tube defects (NTD) is high thus many children are born with a neural tube defect in Addis Ababa, and surgical closure is a commonly performed procedure at the pediatric neurosurgical specialty center.

**Research question:**

The primary aim is to study the outcomes in children undergoing surgical closure of NTDs and to identify risk factors for readmission, complications and mortality.

**Material and methods:**

Single-center prospective study of all surgically treated NTDs from April 2019 to May 2020.

**Results:**

A total of 228 children, mean age 11 days (median 4) underwent surgery during the study period. There were no in-hospital deaths. Perioperatively 11 (4.8%) children developed wound complications, none of them needed surgery and there was no perioperative mortality. The one-year follow-up rate was 62.7% (143/228) and neurological status remained stable since discharge in all. The readmission and reoperation rates were 38 % and 8 % and risk factors for readmission were hydrocephalus (80%) and open defects (88%). Hydrocephalus (P = 0.05) and younger age (P = 0.02) were identified as risk factors for mortality. The wound-related complication rate was 55% at and was associated with large defects (P = 0.04) and delayed closure due to late hospital presentation (P = 0.01).

**Discussion and conclusion:**

The study reveals good perioperative surgical outcome and further need for systematic improvement in treatment and follow-up of NTD patients especially with hydrocephalus. We identified risk factors for wound-related complications, readmission and mortality.

## Highlights

• Ethiopia has a high prevalence of neural tube defects (NTD), and surgical closure is a commonly performed procedure.
• Ethiopia's first prospective study: analyzed 228 children with NTD operations to assess surgical outcomes.
• Acceptable peri-operative outcome (within 30-days after surgery) with minor morbidity and no mortality.
• One-year followup was 62.7%, and the readmission and reoperation rates were 38 % and 8 %.
• Hydrocephalus was identified as a risk factor for readmission, post-operative mortality and morbidity.

## List of abbreviations

NTDs: neural tube defects
GA: gestational age
LMIC: low and middle-income countries
CNS: central nervous system
CSF: cerebrospinal fluid
CT: computer tomography
BUN: blood urea nitrogen
MMC: myelomeningocele
ANC: antenatal care

## 1 Introduction

Neural tube defects (NTDs) are developmental malformations occurring around the 28th day of gestational age (GA). They are caused by failure of processes related to neurulation (Moore and Persaud, 2008). Treating a child born with an NTD requires multidisciplinary care. The condition causes impairment, reduces quality of life and may be life threatening.

A prenatal diagnosis based on obstetric ultrasound examination and maternal serum or amniotic fluid alpha-fetoprotein levels allows for early diagnosis and discussions about treatment (Ghi et al., 2006). However, in low and middle-income countries (LMIC), most of the NTDs are diagnosed at delivery (Xu et al., 2018).

Surgical closure within the first 48 h is the first line of treatment for NTD in order to reduce the risk of infection and further CNS injury (Radcliff et al., 2016). Treatment with broad-spectrum antibiotics is recommended pre-operatively in cases of open defects (Xu et al., 2018). Approximately 85% of children with NTDs develop hydrocephalus (Hubballah and Hoffman, 1987; Miller et al., 1996; Oktem et al., 2008), which is a major cause of morbidity and mortality (Hubballah and Hoffman, 1987; Ammirati and Raimondi, 1987; Yilmaz et al., 2010).

In Addis Ababa, Ethiopia, hospital cohort studies suggest the overall prevalence of NTDs to be between 6 and 12 per 1000 births (Gedefaw et al., 2018; Taye et al., 2016). This is much higher than registered in other countries, thus illustrating that NTDs represent a substantial health burden in Ethiopia. Closure of neural tube defects (NTDs) is common procedure at neurosurgical unite in Ethiopia, however, we know little about the outcomes. A recent retrospective study at our institution revealed high complication and mortality rates (26.1% and 41%) (Getahun et al., 2021). CSF leakage and age < 7 days were the major predisposing factors for complications, whereas hydrocephalus, neonatal age, postoperative wound complications and major neurological deficits were predictors of mortality within the first four years. The study revealed a need for improving NTD surgical care at our center.

In the present study, we aimed to record in-hospital- and up to one year-outcomes of surgically treated NTD children prospectively. In addition, we aimed to identify risk factors for readmission, complications and death. To our knowledge there is no prospective study published so far about the surgical outcome of NTDs in Ethiopia.

## 2 Patients and methods

### 2.1 Inclusion

We prospectively included children who underwent primary NTD closure at Zewditu memorial Hospital from April 2019 to May 2020, provided informed consent was obtained from the parent or caretaker. Children who had any previous surgery, including NTD closure or hydrocephalus treatment before admission and all spinal occult dysraphisms were excluded.

### 2.2 Data recording

A senior neurosurgical resident overseen by a consultant neurosurgeon screened all children who were enrolled into the study using a screening tool (Appendix 1). The caretakers were mothers and fathers in 205 (90.3%) and 21 (9.3%) cases respectively; one child (0.4%) was brought from an orphanage care center. All caretakers agreed to participate. Neurological status was deﬁned by motor power and tone. Power was graded as 1 – absent; 2 - ﬂickering; 3 – horizontal movement; 4 – movement against gravity; and 5 - full power. We defined defects completely covered by normal skin as “closed”, and all others as “open”. We diagnosed hydrocephalus by clinical evaluation and/or by imaging (head CT or ultrasound (US)). The size of the defect was estimated by multiplying length, width and height. Details from the surgical procedures and any adverse events during the hospital stay were recorded.

We scheduled the patients for routine follow-up and study purposes for up to one year after discharge, using a standardized form for data recording.

## 3 Statistics

All data was recorded prospectively and entered into an SPSS spreadsheet. Student's *t*-test was used for continuous, and Chi square test for categorical variables. The level of significance was set at 0.05.

## 4 Ethics

Ethical approval was given from IRB of Addis Ababa Health Bureau (Ref. no. AAHB 7029/227), the college of Health Science, Addis Ababa University (Protocol no. 088/17/Surg) and the Norwegian Ethical Committee for research (REK Vest, project no. 103230). Details of the study were explained to the participants and the caretakers signed consent before participation. Illiterate caretakers gave their oral consent, witnessed by another person.

## 5 Results

### 5.1 Clinical presentation (Table 1)

Table 1 shows baseline data on 228 included children diagnosed with MMC, with a mean age of 11 days and a mean weight of 3975g. Only 64 children were admitted within 48 h after birth. Altogether 210 (92.1%) mothers had received ANC and 156 (74.2%) were taking folate during pregnancy.

**Table 1:** Demography and clinical findings on admission in 228 patients operated with NTD closure.

**Age at presentation in days**	
Mean	11
Median, interquartile range	5 (1–105)
Children Presented with 48 h	66
Children operated within 48 h	64
**Weight in grams**	
Mean	3975.13
Median	3110.00
**Sex**	
Female, n (%)	114 (50%)
**ANC follow-up-Yes, n (%)**	210 (92.1%)
**NTD diagnosed in pregnancy, n (%)**	61 (29%)
**Folate intake during ANC follow-up** - **Yes, n (%)**	156 (74.2%)
**Distance of maternal residence address from the hospital**	
<50 km	58 (25.9%)
50–200 km	46 (20.5%)
200–500 km	111 (49.6%)
500 km	8 (3.6%)
**Hydrocephalus - Yes, n (%)**	63(27.6%)
**Spinal defect size,** Mean (range)SD in mm³	102.8 (61.42–233.5) 56.3
**Motor Power**	
Grade I	108 (49.5%)
Grade 2	7 (3.2%)
Grade 3	5 (2.3%)
Grade 4	2 (0.9%)
Grade 5	96 (44%)
**Anal tone**	
Intact	67 (48.2%)
Decreased	32 (23.0%)
Lax	40 (28.8%)
**Urinary function**	
Normal	98 (46.7%)
Incontinent	35 (16.7%)
Retention	77 (36.7%)

**Table 2:** Follow-up status after 1 years.

Follow-up at one year	n (%)
Responded (% of total)	143 (62.7%)
**Survival status**	
Alive (% of responders)	119 (83.2%)
**Wound Status**	
Wound related Complication	80 (55.9%)
**Hydrocephalus at baseline**	
Within lost to follow-up group	37.6 %
Within follow-up group	22.4 %
**Hydrocephalus on follow-up**	
Yes, n (%)	57/143 (39.9%)
**Re-admission**	
Yes, n (%)	57(143 (40%)

**Table 3:** Predictors of wound complications.

	Wound related complication, n (%)		
	Wound complication (n = 80)	No wound Complication (n = 60)	P value
**Mean age in days during the first admission**	13.0	9.7	0.01^b^
**Type of defect**			
Open	51 (54%)	43 (45%)	0.09^a^
Closed	14 (53%)	12 (46%)	
**Size of the defect, n (mean size in mm³)**	68(162.93)	56(71.19)	0.04^b^
**Pre-operative active CSF leak**			
CSF leak	1 (50%)	1(50%)	0.64^a^
No CSF leak	74 (60%)	50 (40%)	
**Hydrocephalus on follow-up**			
Hydrocephalus	38 (56%)	29 (43%)	0.08^a^
No hydrocephalus	41 (56%)	31 (43%)	

a Chi-square test.

b Student's t-test.

The distribution of defects along the neuraxis was within the same range as the retrospective material previously published by us; 6 % encephalic, 0.5 % cervical, 11.1% thoracic, 82.2 % lumbar or sacral. The mean size of the spinal defects was 102.8 mm³, (range 61.4–233.5). Skin covering status was documented in 186 children; 143 defects (76.9%) were open and 43 (23.1%) were closed. All open defects were in the lumbar area, and six of these had ongoing CSF leakage.

Altogether 63 (27.6%) children were diagnosed with hydrocephalus on admission. CT or ultrasound of 147 children revealed hydrocephalus in 50; another 13 developed symptoms of hydrocephalus before MMC closure.

A total of 108 children (49.5%) had paraplegia, while 96 (44%) were neurologically intact. Paraplegia and/or urinary retention or incontinence was found exclusively in cases with lumbar or mid/lower thoracic defects (61.5% and 24.5% of cases per level, respectively).

Among 210 examined children, 112 (53.3%) had urinary retention or incontinence.

Nine children had ongoing infections on admission, including pneumonia and meningitis; these were treated before MMC closure. Congenital malformations; clubfoot, cardiac and renal malformations were seen in 24 cases; all of these received defect closure during the primary admission.

The mean pre-operative stay in the hospital was 9 days (median 5).

### 5.2 MMC closure

The defects were closed by standard multilayer technique under general anaesthesia and antibiotic covering; the surgeon (a consultant or a senior resident under supervision) used magnifying glasses. Three cases received a rotational skin flap to obtain closure. We obtained information about intra-operative findings for 194 children. Neural tissue was identified inside the sac in 167 defects.

### 5.3 Hydrocephalus treatment

Twenty-nine out of the 64 hydrocephalic children received a VP shunt during their primary hospital stay, 12 children out of 29 got their shunts before MMC closure due to severe symptoms of hydrocephalus. The remaining 35 children with a radiological diagnosis of hydrocephalus but no symptoms were discharged with a follow-up appointment.

### 5.4 Perioperative complications

During the first 30 days after MMC closure, 11 (4.8%) children developed wound complications; 9 had superficial wound infections and 2 had wound dehiscence which were treated with oral antibiotics and honey wound dressings. All 228 children were discharged alive.

### 5.5 1 year post-operative follow-up (Table 2)

Altogether 143 (62.7%) out of 228 children were either brought for one-year follow up, or we obtained information from caretakers via telephone. For the remaining 85, caretakers did not respond to repeated telephone calls. Among the 143, 119 (83.2%) were alive and 24 (16.8%) were dead. We compared the lost-to-follow-up group with the 143 cases on baseline parameters, and found a significant higher proportion of cases with hydrocephalus (37.6 % vs 22.4 %, p = 0.05) among those who were lost.

### 5.6 Neurological function

Among 143 children, a postoperative neurological status during the first year was available for 139, and there was no difference compared to preoperative. Out of 139, 66 children displayed crawling or ambulatory abilities with full motor power, while 60 children experienced complete paralysis. The remaining 13 children exhibited motor power ranging from Grade II to IV. Among these 139, 100 had bladder dysfunction. Caretakers performed regular clean intermittent catheterization (CIC) using pediatric nasogastric feeding tubes for 68 of these, whereas 32 with bladder dysfunction did not receive CIC.

### 5.7 Hydrocephalus

We retrieved data for 12 out of the 29 children who received a VP shunt during primary admission. One child was deceased, and 5 received a revision within the first year. Among 35 children who radiologically had hydrocephalus on primary admission and were discharged without treatment of hydrocephalus, follow-up data was available for 16, and all received a shunt later. The remaining 19 children did not return for follow-up, leading to a loss of follow-up of 54% among children with hydrocephalus.

Another 47 children developed radiological hydrocephalus during the first year and 29 of them had symptoms and received a shunt. Thus, altogether 57 out of 143 children (39.9%) received a shunt within one year.

### 5.8 Wound related complications after discharge (Table 3)

In total, 80 children (55.9%) experienced complications after discharge, including CSF leak, superficial wound infections, wound dehiscence and pseudomeningocele. Only five of them needed surgery (CSF leak repair – 3, External ventricular drainage – 1, Wound revision – 1), the rest were treated with antibiotics and wound care.

Older age at presentation (p = 0.01), and larger defect size (p = 0.04) were significantly associated with wound related complications.

### 5.9 Readmissions and reoperations (Table 4)

During the follow-up period, 57 children (40%) were re-admitted; 35 for a new VP-shunt insertion and 11 for conditions requiring a reoperation (CSF diversion procedures and wound revisions). Readmission rates were comparatively higher in children with hydrocephalus (p = 0.02) and children with open defects (p = 0.05).

### 5.10 One-year mortality (Tabel 5)

The one-year mortality rate was 24/143 (16.8%). The causes of death were hydrocephalus, meningitis, pneumonia, sepsis, and shunt infections. Hydrocephalus and young age were associated with higher mortality rates (p = 0.05 and 0.02, respectively) (Table-5).

**Table 4:** Risk factors for re-admission.

	Re-admission		
	Re-admission (n = 57)	No Re-admission (n = 87)	P value
**Preoperative Hydrocephalus**			
Hydrocephalus	18 (32%)	13 (16%)	0.02^a^
No Hydrocephalus	38 (68%)	70 (84%)	

**Mean age, n (age in days during the first admission**)	54 (11.2)(2 missing)	82 (11.8)(5 missing)	0.8^b^
**Type of defect**			
Open	37 (88%)	57 (74%)	0.05^a^
Closed	5 (12%)	20 (26%)	
	(14 missing)	(10 missing)	
**Size of the defect, n (mean size in mm³)**	43 (67.2) (13 missing)	80 (152) (7 missing)	0.08^b^

a Chi-square test.

b Student's t-test.

**Table-5:** Predictors of mortality.

	Survival Status, n (%)		
	Alive	Dead	P value
**Hydrocephalus on followup**			
Hydrocephalus	54 (79%)	14 (20%)	0.05^1^
No Hydrocephalus	65 (90%)	7 (9.7%)	
**Mean age, n (age in days during the first admission)**	116(12.1)	24(9.42)	0.02^2^
**Type of defect**			
Open	78(81.2%)	18(18.7%)	0.07^1^
Closed	24(92.3%)	2(7.6%)	
**Wound status on 1 year follow-up**			
Wound complication	47(78.3%)	13(21.7%)	0.08^1^
No complication	72(90%)	8(10%)	
**Re-admission status**			
Re- admission	87(84.5%)	16(15.5%)	0.07^1^
No re- admission	32(88.8%)	4(11.1%)	
**Re-operation status**			0.07
Re- operation, shunt revision	4(80%)	1(20%)	
Re- operation, EVD insertion	0	2(100%)	
**Neurological function**			0.08
Absent motor power	119(85%)	20(14%)	

## 6 Discussion

### 6.1 Aim of the study

Closure of neural tube defects (NTDs) in children is a common neurosurgical procedure in Ethiopia. We published a retrospective study of surgical outcomes of NTD-children operated six years ago at the pediatric neurosurgical center in Addis Ababa (Getahun et al., 2021). Compared with previous, children received treatment earlier (mean age 11 vs 243 days), and a higher proportion had an open defect (76% vs 14 %). The mortality rate (16.7% at one-year vs 41% at four years) was lower, but a comparison was not feasible (Table 6).

**Table 6:** Comparison between the retrospective (2014) and prospective studies (2020).

	Retrospective study, 2014 operations	Prospective study, 2020 operation
n	n = 88	n = 228
Age in days during the first admission, mean (median) range	243 (30) 1-3600	11 (5) 1-105
Patients presented < 48 h after birth	12 (13.6%)	64 (29.1%)
Open defects, n (%)	13 (14.8%)	143 (76.8%)
Closed defects, n (%)	75 (85.2%)	43 (23.11%)
Morbidity rate(wound related complications) n (%)	23(26.1%)	80 (55.8%)
Mortality rate, n (%)	25(41%) (after 4 years)	24 (16.7%) (after a year)
Risk factors for morbidity	Age < 7 days	Younger age
	Pre-operative CSF leak	Having large size of the defect
		More intra-operative bleeding
Risk factors for mortality	Age < 30 days	Hydrocephalus
	Preoperative hydrocephalus	Younger age
	Preoperative absent or incomplete motor function	
	Postoperative wound related complications	

During the last decade, access to neurosurgical services in different part of Ethiopia has greatly improved, and there has been a buildup of resources at the center (Asfaw et al., 2021; Ethiopia Demographic and Health Survey, 2012). Thus, an update study with a prospective design was indicated.

### 6.2 Population and ANC

Altogether 75% of children came from outside Addis Ababa. Transportation from rural areas in Ethiopia takes time, but children came to treatment earlier than those operated six years ago. This can be due to improvement in public awareness, health-seeking behavior, and neurosurgical capacity and transportation facilities.

The study further reveals a significant rise in the proportion of children with open defects and the percentage is now in line with other published data (Sahni et al., 2022). Untreated, open defects are associated with high mortality rates, so we assume that many of the affected children died untreated earlier.

Even though most of the mothers had ANC follow-up, NTDs were detected prenatally only in 29% of cases. ANC service in health centers involves personnel with limited competence and ultrasound equipment may be either inadequate or unavailable. The study as the retrospective study reveals the importance of better ANC diagnosis and follow-ups.

### 6.3 Surgical outcome

#### 6.3.1 Complications

The study reveals a reduction in perioperative complications rate compared to the previous. Although a high number of children returned for a wound related complication, the vast majority of those were minor. Surgeons involved in the study face a large caseload and they are highly trained in performing MMC closure. As large defects was a risk factor for complications, we assume that refining closure techniques including the use of rotational flaps may bring down the complication rates further. In contrast to our retrospective study, a preoperative CSF leak did not increase wound infection rates, probably because closure was done earlier than before. A recently published study form Zambia revealed 31% complication- and 7% mortality rates within 30 days (Reynolds et al., 2021).

#### 6.3.2 Hydrocephalus

Current recommendations suggest that children with hydrocephalus associated with NTDs undergo one-stage surgery within 48 h of birth. (Thompson, 2009; Giné et al., 2018). If the shunt is inserted before defect closure, the shunt infection rate increases (McDowell et al., 2018a). However, in our study, 12 children with severe hydrocephalus got treatment with VP shunts before the MMC closure as they were assessed unsuitable candidates for simultaneous NTD closure and ventriculoperitoneal (VP) shunt insertion during a single surgery. We do assume that in our cases, especially with open defects, there may be colonization of bacteria. There were many children with hydrocephalus lost to follow-up; we assume many of them had died. Timing of hydrocephalus treatment is mandatory especially with open defects and reports advocate that the timing of hydrocephalus surgery should preferably be after the MMC closure in order to avoid complications (Melekoglu et al., 2016; Kshettry et al., 2014; Kshettry et al., 2014, 2014; Seitzberg et al., 2008). 35 children with radiological hydrocephalus were discharged without shunting and of these 15 needed shunt on follow-up and 19 were lost to follow-up. Our decision not to perform immediate shunt insertion in children with asymptomatic hydrocephalus was based on careful clinical evaluation and consideration of factors such as the absence of clinical symptoms, stability of the patient's condition, and the potential risks and benefits associated with early shunt intervention. These findings suggest the need for further evaluation of cases with radiological evidence of hydrocephalus but without symptoms. We conclude that in the future, it would be beneficial to conduct a more careful evaluation of each case with radiological evidence of hydrocephalus before discharge, and to provide thorough information and counseling to parents in such cases.

#### 6.3.3 Readmission

We had a readmission rate of 40.3%, however, the reoperation rate was lower (7.7%). A similar report from a middle-income country found 24% readmissions, mainly due to wound dehiscence and purulence (McDowell et al., 2018b). Most of the readmissions in our study (61.4%) were due to shunt-requiring hydrocephalus. There was a high (76.8%) proportion of open defects in our study, and other studies have showed that open defects are more prone to have infections (Idris, 2011; Sims-Williams et al., 2017). The results may emphasize the importance of including swabs for bacteriological examination pre-operatively and proper treatment of infected defects before surgery.

#### 6.3.4 Mortality

The mortality rate was 17% at one-year, but as we did not register one-year mortality in the previous study, the rates cannot be compared. Hydrocephalus and younger age were risk factors for mortality in both studies. The mortality rate in our population is in line with a report from another Sub-Saharan country (Xu et al., 2018). There is room at our center for improvement in per-and postoperative care which likely will lead to a further decrease in mortality (Idris, 2011).

## 7 Conclusion

Although a statistical comparison with the previous study was not feasible, outcomes seem to have improved, but readmission and mortality rates are still high. A large defect and a delay in presentation were risk factors for wound-related complications, which were minor in all except a few. Hydrocephalus and open defect were risk factors for readmission. Hydrocephalus and young age were associated with a high mortality rate.

Despite the higher prevalence of NTDs in Ethiopia, there is a need for improved postnatal care for affected children. Advanced treatments such as intrauterine surgery, as demonstrated by the MOMS trial, could be beneficial. However, in low-income countries like Ethiopia, there is a requirement for enhancement in healthcare delivery, as prenatal detection of NTDs remains significantly lower. This represents an area where partnerships, professional collaborations, and knowledge exchange between HICs and LMICs could contribute to enhancing global care for children with NTDs.

## 8 Limitations

The main limitation of the study is the low follow-up rate. This brings uncertainty to the mortality rate, and we may assume that many caretakers did not show up because the child was dead. These limitations are common in LICs, where communication is difficult due to geographic and communication-related obstacles.

## Funding

10.13039/501100007941Addis Ababa University, Ethiopia funded planning of the project and the data collection. 10.13039/501100005036University of Bergen, Norway granted a PhD position for the first author.

## Disclosure-conflict of interest statement

The authors whose names are listed below certify that they have no affiliations with or involvement in any organization or entity with any financial interest (such as honoraria; educational grants; participation in speakers’ bureaus; membership, employment, consultancies, stock ownership, or other equity interest; and expert testimony or patent-licensing arrangements), or non-financial interest (such as personal or professional relationships, affiliations, knowledge or beliefs) in the subject matter or materials discussed in this manuscript.

## References

[bib1] Ammirati M., Raimondi A.J. (1987). Cerebrospinal fluid shunt infections in children. A study on the relationship between the etiology of hydrocephalus, age at the time of shunt placement, and infection rate. Childs Nerv Syst.

[bib2] Asfaw Z.K., Tirsit A., Barthélemy E.J., Mesfin E., Wondafrash M., Yohannes D., Ashagre Y., Park K., Laeke T. (2021). Neurosurgery in Ethiopia: a new chapter and future prospects. World Neurosurg.

[bib3] (2012). Ethiopia Demographic and Health Survey.

[bib4] Gedefaw A., Teklu S., Tadesse B.T. (2018). Magnitude of neural tube defects and associated risk factors at three teaching hospitals in Addis Ababa, Ethiopia. BioMed Res. Int..

[bib5] Getahun S., Masresha S., Zenebe E., Laeke T., Tirsit A. (2021). Four-year treatment outcomes of children operated for neural tube defect in Addis Ababa, Ethiopia: a retrospective study. World Neurosurg.

[bib6] Ghi T., Pilu G., Falco P., Segata M., Carletti A., Cocchi G., Santini D., Bonasoni P., Tani G., Rizzo N. (2006). Prenatal diagnosis of open and closed spina bifida. Ultrasound Obstet. Gynecol..

[bib7] Giné C., Arévalo S., Maíz N., Rodó C., Manrique S., Poca A., Molino J.A., Carreras E., López M. (2018). Fetoscopic two-layer closure of open neural tube defects. Ultrasound Obstet. Gynecol..

[bib8] Hubballah M.Y., Hoffman H.J. (1987). Early repair of myelomeningocele and simultaneous insertion of ventriculoperitoneal shunt: technique and results. Neurosurgery.

[bib9] Idris B. (2011). Factors affecting the outcomes in children post-myelomeningocoele repair in northeastern peninsular Malaysia. Malays. J. Med. Sci..

[bib10] Kshettry V.R., Kelly M.L., Rosenbaum B.P., Seicean A., Hwang L., Weil R.J. (2014). Myelomeningocele: surgical trends and predictors of outcome in the United States, 1988-2010. J. Neurosurg. Pediatr..

[bib11] McDowell M.M., Lee P.S., Foster K.A., Greene S. (2018). The use of external ventricular drainage to reduce the frequency of wound complications in myelomeningocele closure. Pediatr. Neurosurg..

[bib12] McDowell M.M., Lee P.S., Foster K.A., Greene S. (2018). The use of external ventricular drainage to reduce the frequency of wound complications in myelomeningocele closure. Pediatr. Neurosurg..

[bib13] Melekoglu R., Eraslan S., Celik E., Simsek Y. (2016). Perinatal and neonatal outcomes of patients who were diagnosed with neural tube defect in midtrimester fetal ultrasound scan and refused request for termination of pregnancy. BioMed Res. Int..

[bib14] Miller PD, Pollack IF, Pang D, Albright AL. Comparison of simultaneous versus delayed ventriculoperitoneal shunt insertion in children undergoing myelomeningocele repair. J. Child Neurol.. 1996 Sep;11(5):370-372. doi: 10.1177/088307389601100504.PMID:8877603. 10.1177/088307389601100504 8877603

[bib15] Moore K.L., Persaud T.V.N. (2008).

[bib16] Oktem I.S., Menkü A., Ozdemir A. (2008). When should ventriculoperitoneal shunt placement be performed in cases with myelomeningocele and hydrocephalus?. Turk Neurosurg.

[bib17] Radcliff E., Cassell C.H., Laditka S.B., Thibadeau J.K., Correia J., Grosse S.D., Kirby R.S. (2016). Factors associated with the timeliness of postnatal surgical repair of spina bifida. Childs Nerv Syst.

[bib18] Reynolds R.A., Bhebhe A., Garcia R.M., Chen H., Bonfield C.M., Lam S., Sichizya K., Shannon C. (2021). Surgical outcomes after myelomeningocele repair in lusaka, Zambia. World Neurosurg.

[bib19] Sahni M., Alsaleem M., Ohri A. (2022). StatPearls.

[bib20] Seitzberg A., Lind M., Biering-Sørensen F. (2008). Ambulation in adults with myelomeningocele. Is it possible to predict the level of ambulation in early life?. Childs Nerv Syst.

[bib21] Sims-Williams H.J., Sims-Williams H.P., Kabachelor E.M., Fotheringham J., Warf B.C. (2017). Ten-year survival of Ugandan infants after myelomeningocele closure. J. Neurosurg. Pediatr..

[bib22] Taye M., Afework M., Fantaye W., Diro E., Worku A. (2016). Magnitude of birth defects in central and northwest Ethiopia from 2010-2014: a descriptive retrospective study. PLoS One.

[bib23] Thompson D.N. (2009). Postnatal management and outcome for neural tube defects including spina bifida and encephalocoeles. Prenat. Diagn..

[bib24] Xu L.W., Vaca S.D., He J.Q., Nalwanga J., Muhumuza C., Kiryabwire J., Ssenyonjo H., Mukasa J., Muhumuza M., Grant G. (2018). Neural tube defects in Uganda: follow-up outcomes from a national referral hospital. Neurosurg. Focus.

[bib25] Yilmaz A., Müslüman A.M., Dalgic N., Cavuşoğlu H., Kanat A., Colak I., Aydın Y. (2010). Shunt insertion in newborns with myeloschisis/myelomenigocele and hydrocephalus. J. Clin. Neurosci..

