# Supplementary material for: Surgical treatment outcome of children with neural-tube defect: A prospective cohort study in a high volume center in Addis Ababa, Ethiopia
Source: Brain Spine. 2023 Jul 26;3:101787. doi: 10.1016/j.bas.2023.101787 (PMC10668049; doi:10.1016/j.bas.2023.101787)
Supplement: Multimedia component 1 [file mmc1.docx]

**Appendix 1**

| Age in days (on Admission) |  |
| --- | --- |
| Sex |  |
| Weight at operation in grams |  |
| Maternal age during delivery |  |
| Residential address |  |
| Site of the Swelling  over the back  posterior aspect of the head  anterior aspect of the head |  |
| Discharge from the swelling |  |
| History of previous pregnancy complicated with NTDs |  |
| Parity of the mother |  |
| ANC follow-up during pregnancy |  |
| Head circumference |  |
| Size of the swelling |  |
| CSF leak |  |
| Super-infection |  |
| Presence of associated kyphosis |  |
| Other congenital malformation |  |
| Hemoglobin |  |
| Blood urea nitrogen |  |
| Creatinine |  |
| Abdominal ultrasound |  |
| Brain ultrasound/CT-scan |  |
| Intra-op findings |  |
| Bleeding volume |  |
| Injury to vital structures |  |
| Anesthesia related complication |  |
| Hydrocephalus clinical or imaging |  |
| Post-op CSF leakage from the wound |  |
| Deterioration of neurological status |  |
| Wound related complication |  |
| Clinical finding |  |
| Complication / reoperation on follow-up |  |
| Neurological status |  |
| Hydrocephalus on follow up |  |
